# Chlorophyllin Supplementation of Medicated or Unmedicated Swine Diets Impact on Fecal *Escherichia coli* and Enterococci

**DOI:** 10.3390/ani14131955

**Published:** 2024-07-02

**Authors:** Kristina M. Feye, Mark A. Rasmussen, Kathleen M. Yeater, Robin C. Anderson, Tawni L. Crippen, Roger B. Harvey, Toni L. Poole, Steven C. Ricke

**Affiliations:** 1 Cell and Molecular Biology Program, University of Arkansas, Fayetteville, AR 72701, USA; 2 Department of Animal Science, Iowa State University, Ames, IA 50011, USA; markras8888@hotmail.com; 3 United States Department of Agriculture/Agricultural Research Service, Plains Area Office of the Director, Fort Collins, CO 80521, USA; kathleen.yeater@usda.gov; 4 Southern Plains Agricultural Research Center, United States Department of Agriculture/Agricultural Research Service, College Station, TX 77845, USA; robin.anderson@usda.gov (R.C.A.); tc.crippen@usda.gov (T.L.C.); roger.harvey@usda.gov (R.B.H.); toni.poole@usda.gov (T.L.P.); 5 Meat Science and Animal Biologics Discovery Program, Department of Animal and Dairy Sciences, University of Wisconsin, Madison, WI 53706, USA

**Keywords:** antimicrobial resistance, chlorophyll metabolites, efflux pump inhibitors, medicated feed, zoonotic pathogens

## Abstract

**Simple Summary:**

Feeding pigs diets containing added antibiotics may select for a high carriage of bacterial genes coding for multidrug efflux pumps that can contribute to antibiotic resistance. It has recently been demonstrated that the digestion of chlorophyll in the gut of animals consuming green forage can yield compounds that inhibit efflux pump activity. Considering that commercially reared pigs generally do not eat substantial amounts of feeds containing chlorophyll, this study was conducted to test if feeding pigs a water-soluble chlorophyll product could affect the antibiotic resistance profiles of wild-type populations of fecal bacteria such as *Escherichia coli* and enterococci. Results from two feeding trials indicate that feeding chlorophyll as a source or precursor of some known efflux pump inhibitors may indeed promote a decrease in antibiotic resistance in the enterococci but not the *Escherichia coli*. However, under the conditions of the present study, the decrease in enterococcal resistance was likely not enough to warrant development into an inexpensive technology to preserve and enhance the efficacy of currently available antibiotics. However, further research designed to optimize chlorophyll administration could potentially lead to practical applications for the swine industry.

**Abstract:**

Considering that certain catabolic products of anaerobic chlorophyll degradation inhibit efflux pump activity, this study was conducted to test if feeding pigs a water-soluble chlorophyllin product could affect the antibiotic resistance profiles of select wild-type populations of fecal bacteria. Trial 1 evaluated the effects of chlorophyllin supplementation (300 mg/meal) on fecal *E. coli* and enterococcal populations in pigs fed twice daily diets supplemented without or with ASP 250 (containing chlortetracycline, sulfamethazine and penicillin at 100, 100 and 50 g/ton, respectively). Trial 2, conducted similarly, evaluated chlorophyllin supplementation in pigs fed diets supplemented with or without 100 g tylosin/ton. Each trial lasted 12 days, and fecal samples were collected and selectively cultured at 4-day intervals to enumerate the total numbers of *E. coli* and enterococci as well as populations of these bacteria phenotypically capable of growing in the presence of the fed antibiotics. Performance results from both studies revealed no adverse effect (*p* > 0.05) of chlorophyllin, antibiotic or their combined supplementation on average daily feed intake or average daily gain, although the daily fed intake tended to be lower (*p* = 0.053) for pigs fed diets supplemented with tylosin than those fed diets without tylosin. The results from trial 1 showed that the ASP 250-medicated diets, whether without or with chlorophyllin supplementation, supported higher (*p* < 0.05) fecal *E. coli* populations than the non-medicated diets. Enterococcal populations, however, were lower, albeit marginally and not necessarily significantly, in feces from pigs fed the ASP 250-medicated diet than those fed the non-medicated diet. Results from trial 2 likewise revealed an increase (*p* < 0.05) in *E. coli* and, to a lesser extent, enterococcal populations in feces collected from pigs fed the tylosin-medicated diet compared with those fed the non-medicated diet. Evidence indicated that the *E. coli* and enterococcal populations in trial 1 were generally insensitive to penicillin or chlortetracycline, as there were no differences between populations recovered without or with antibiotic selection. Conversely, a treatment x day of treatment interaction observed in trial 2 (*p* < 0.05) provided evidence, albeit slight, of an enrichment of tylosin-insensitive enterococci in feces from the pigs fed the tylosin-medicated but not the non-medicated diet. Under the conditions of the present study, it is unlikely that chlorophyllin-derived efflux pump inhibitors potentially present in the chlorophyllin-fed pigs were able to enhance the efficacy of the available antibiotics. However, further research specifically designed to optimize chlorophyll administration could potentially lead to practical applications for the swine industry.

## 1. Introduction

Public health officials and many in the general public are concerned that the agricultural use of antibiotics is contributing to the emergence of antimicrobial-resistant bacteria which may decrease the effectiveness of antimicrobials important in human medicine [1,2]. The acquisition of resistance by microorganisms on the farm may also decrease the effectiveness of the limited number of antibiotics currently available to producers [3]. For instance, Looft et al. reported that the gut microbiome of pigs fed the antibiotic supplement ASP 250 (containing chlortetracycline, sulfamethazine and penicillin) maintained higher *Escherichia coli* concentrations than pigs fed unmedicated diets and also had a higher carriage of antimicrobial resistance genes [4]. A high carriage of genes coding for multidrug efflux pumps was observed within the pig gut microbiome after the feeding of ASP 250; however, the carriage of these genes was high even before administration of the ASP 250 product [4]. Selection for a sustained resistance to tylosin, an antibiotic often used more during the growing and finishing phases of swine than ASP 250, was observed with fecal enterococcal populations during a 35-day tylosin administration period to finishing swine [5]. In this case, a transient increase in the prevalence of the *erm*(B) was observed from day 0 to day 21 of treatment but then declined to near initial levels by day 35, suggesting that the sustained resistance was conferred by mechanisms other than that expressed by *erm*(B) [5]. Multidrug efflux pumps confer resistance to bacteria by enabling them to actively excrete or pump antibiotics as well as some toxic chemical agents of their cytoplasm [6]. Multidrug efflux pumps can be categorized into the following five main types or families of membrane-located transport systems: (1) ATP-binding cassette (ABC) transporters, (2) small multidrug resistance (SMR), (3) resistance–nodulation–division (RND), (4) major facilitator superfamily (MF) and (5) the multiple antibiotic and toxin extrusion (MATE) systems [7]. These systems are widely distributed among numerous bacterial pathogens and can oftentimes be carried in combination within a single bacterial species. In *Salmonella* and *E. coli*, the AcrAB efflux pump which is a member of the RND family confers the ability to excrete fluoroquinolones, tetracyclines, chloramphenicol, and novobiocin, and it can contribute to resistance to some β-lactam antibiotics and erythromycin [8,9].

Numerous naturally occurring plant compounds and their anaerobic degradation products, such as the chlorophyll catabolite pheophorbide-a, inhibit efflux pumps of Gram-positive and Gram-negative bacteria similar to that by a synthetic inhibitor, l-phenylalanyll-arginyl-β-naphthylamide (PAβN) [10]. This can lead to resistant bacteria becoming 10- to 100-fold more susceptible to several different antibiotics [11,12]. Pheophorbide-a or other degradation products of chlorophyll or chlorophyll derivatives, such as the water-soluble derivative, chlorophyllin, can accumulate in the gut of animals consuming green plant material [13,14,15]. Less is known about the fate of ingested chlorophyllin than ingested chlorophyll in food animals, although some reports suggest that their digestion products may be structurally similar to those of natural chlorophylls with the exception that the copper or zinc cations may be retained within the porphyrin ring, yielding products such as copper pheophytin and copper-protoporphyrin, whereas magnesium is usually from the lost porphyrin ring during the early stages of digestion [16,17,18,19]. Therefore, we hypothesized that feeding chlorophyll to commercially reared pigs, which normally consume little if any chlorophyll, may also promote the gut accumulation of chlorophyll degradation products. If such an event were to occur, it seems reasonable to further speculate that the accumulation of degradation products such as pheophorbide in the pig gut may make their microbiota more sensitive to important antimicrobials. Accordingly, the objective of this proposal was to test the effects of supplementing pig diets with a water-soluble chlorophyll product prepared via the substitution of copper for the typical magnesium core ion, on intake average daily gain and antimicrobial sensitivity of *E. coli* and enterococci to the feed grade antibiotics penicillin, chlortetracycline and tylosin.

## 2. Methods

### 2.1. Animals

Male and female progeny from Landrace × Yorkshire dams crossed with Pietrain × Duroc × Hampshire sires were obtained from a local producer. Upon delivery to the swine-rearing facilities at the USDA Southern Plains Agricultural Research facility, all pigs were cared for according to a protocol approved by the Center’s Institutional Animal Care and Use Committee (ACUC Protocol #2104005 approved 15 October 2014). The present study was with two separate phases conducted one after the other to evaluate the effect of non-medicated or medicated diets supplemented without or with water-soluble chlorophyllin on wild-type *E. coli* and enterococci within the competitive porcine gut environment during the feeding starter or growing diets (Table 1). Chlorophyllin was purchased from MP Biologicals LLC. (Solon, OH, USA). penicillin (Penicillin G), chlortetracycline and tylosin (tylosin-tartrate) were all purchased from Sigma (St. Louis, MO, USA).

### 2.2. Phase 1, Effects of Non- and ASP 250-Medicated (Chlortetracycline, Penicillin and Sulfamethazine) Diets without or with Chlorophyllin Supplementation on Fecal E. coli and Enterococcal Populations

Accordingly, during Phase 1, 21 pigs (averaging 69 ± 9.8 kg) were each randomly placed into 1 of 21 available pens and acclimated for two weeks to a non-medicated starter diet (Table 1). Thereafter, each pen, with its individual pig, was assigned to the following treatments: non-medicated without added chlorophyllin (*n* = 5 pigs), non-medicated plus added chlorophyllin (*n* = 4 pigs), ASP 250-medicated without added chlorophyllin (*n* = 6 pigs) and ASP 250-medicated plus added chlorophyllin (*n* = 6 pigs). Phase 1 diets were fed for 12 days. The ASP 250-medicated diet contained chlortetracycline, sulfamethazine and penicillin (100, 100 and 50 g/ton, respectively).

### 2.3. Phase 2, Effects of Non- and Tylosin-Medicated Diets without or with Chlorophyllin Supplementation on Fecal E. coli and Enterococcal Populations

During Phase 2, which commenced immediately after Phase 1, the pigs were switched from the ASP 250-medicated starter diets to tylosin-medicated grower diets (Table 1) so that the Phase 2 treatments were non-medicated without added chlorophyllin (*n* = 5 pigs), non-medicated plus added chlorophyllin (*n* = 4 pigs), tylosin-medicated without added chlorophyllin (*n* = 6 pigs) and tylosin-medicated plus added chlorophyllin (*n* = 6 pigs). Tylosin was included at 100 g/ton during the Phase 2 trial, and dietary treatments were fed for 12 days. 

### 2.4. Diet Administration, Feed Refusals and Pig Weighing

The diets were fed individually to the pigs, each housed in a separate pen, twice daily (07:30 and 16:30) by first offering a 20% portion of the meal. To promote total consumption of the chlorophyllin treatment (300 mg per each meal), it was top dressed on the feed and offered individually to all pigs fed the chlorophyllin-supplemented diets. Approximately 30 min after the initial feeding, the remainder of the meal was offered to provide 1.5 times each meal’s expected dry matter intake. Feed refusals from each meal were recovered prior to offering of the next meal, cleaned of any observable fecal material, dried at 100 °C and weighed to allow the determination of daily intakes as the difference between dry matter offered and dry matter recovered. The pigs not receiving the chlorophyllin product were similarly fed each meal in two portions. Pigs were weighed at the beginning and end of each phase during study 1 and days 0, 12 and 24 of study 2, and average daily gains were calculated as the increase in weight divided by 12 days. 

### 2.5. Bacteriological Evaluations

For each trial, fresh fecal contents were collected after the morning feeding at 4-day intervals beginning at the start of each feeding period and continued until the end of the study 24 days later. The fecal contents were returned to the lab and cultured on BBL™ MacConkey Agar MacConkey agar and on Difco™ m *Enterococcus* agar (Becton Dickinson Microbiology Systems, Sparks, MD, USA) each supplemented without or with 8 µg penicillin/mL, 16 µg chlortetracycline/mL or with 100 µg tylosin/mL to enumerate total generic *E. coli* and enterococcal populations as well as those phenotypically capable of growing in the presence of the added antibiotic exhibit. Fecal contents sampled prior to the beginning of the experiment were also qualitatively cultured for the presence of *Salmonella* via enrichment on 24 h tetrathionate broth and subsequent plating to Brilliant Green agar (Oxoid Ltd., Basingstoke, Hampshire, England), but *Salmonella* colonies were not observed after 24 h incubation. Consequently, fecal contents were not cultured for *Salmonella* during subsequent collections.

### 2.6. Statistics

The main effects of dietary treatment on performance variables during each feeding trial (ASP 250 during Phase 1 or tylosin during Phase 2) without or with chlorophyllin supplementation were analyzed independently for the main effects of diet using a one-way analysis of variance. Colonies enumerated differential and selective agar media were log_10_ transformed for statistical analysis and are expressed as log_10_ colony-forming units/g (CFU/g) of fecal material. 

A two-way repeated measures analyses of variance was employed to examine changes in the bacterial population responses in individual pig subjects fed differing diets during each 12-day period (measured at start 0, day 4, day 8, and day 12). Phase 1 diets were non-medicated starter diet without added chlorophyllin, non-medicated diet plus added chlorophyllin, ASP 250-medicated starter diet without added chlorophyllin and ASP 250-medicated starter diet plus added chlorophyllin. Phase 2 diets were non-medicated grower diet without added chlorophyllin, non-medicated grower diet plus added chlorophyllin, tylosin-medicated grower without added chlorophyllin and tylosin-medicated grower plus added chlorophyllin. Phase 1 and Phase 2 trials were analyzed independently. Analysis of the repeated measures ANOVA used the GLIMMIX Procedure in SAS/STAT 15.1 © SAS Institute, Inc., Cary, NC, USA with the Auto-Regressive (1) covariance structure and the Laplace method. Differences of least squares means were achieved via the LSMEANS statement with Tukey–Kramer adjusted *p*-values and the LINES option. Least squares means are presented for the significant as well as nonsignificant diet by day on diet interactions for consistency’s sake. When the diet by day on diet interactions were overtly nonsignificant, the least squares means for the main effects of diet and of day on diet are also presented. Correlations and probabilities were generated in JMP 15.1, Multivariate analytical process.

## 3. Results and Discussion

### 3.1. Study 1, Medicated Diet Effects on Feed Intake and Performance

The average daily feed intake did not differ between dietary treatments during Phase 1 or Phase 2; however, average daily intake tended (*p* = 0.0528) to be lower in pigs fed the tylosin-medicated diets (Table 2). Average daily gains did not differ between dietary treatments during Phase 1 or Phase 2 (Table 2). These results indicate that pigs could be safely fed as much as 600 mg of the water-soluble chlorophyllin per pig per day without negative impacts on feed intake or performance. By comparison, Martins et al. have reported little to no negative effects of feeding pigs diets containing as much as 5 ppm total chlorophyll contained within diets supplemented with chlorophyll-containing microalgae (*Arthrospira platensis* or *Chlorella vulgaris*) [19,20]. To our knowledge, there are few if any studies evaluating the effects of chlorophyllin on the performance of pigs. 

### 3.2. Phase 1, Effects of Non- and ASP 250-Medicated (Chlortetracycline, Penicillin and Sulfamethazine) Diets without or with Chlorophyllin Supplementation on Fecal E. coli and Enterococcal Populations

**Figure 1:**
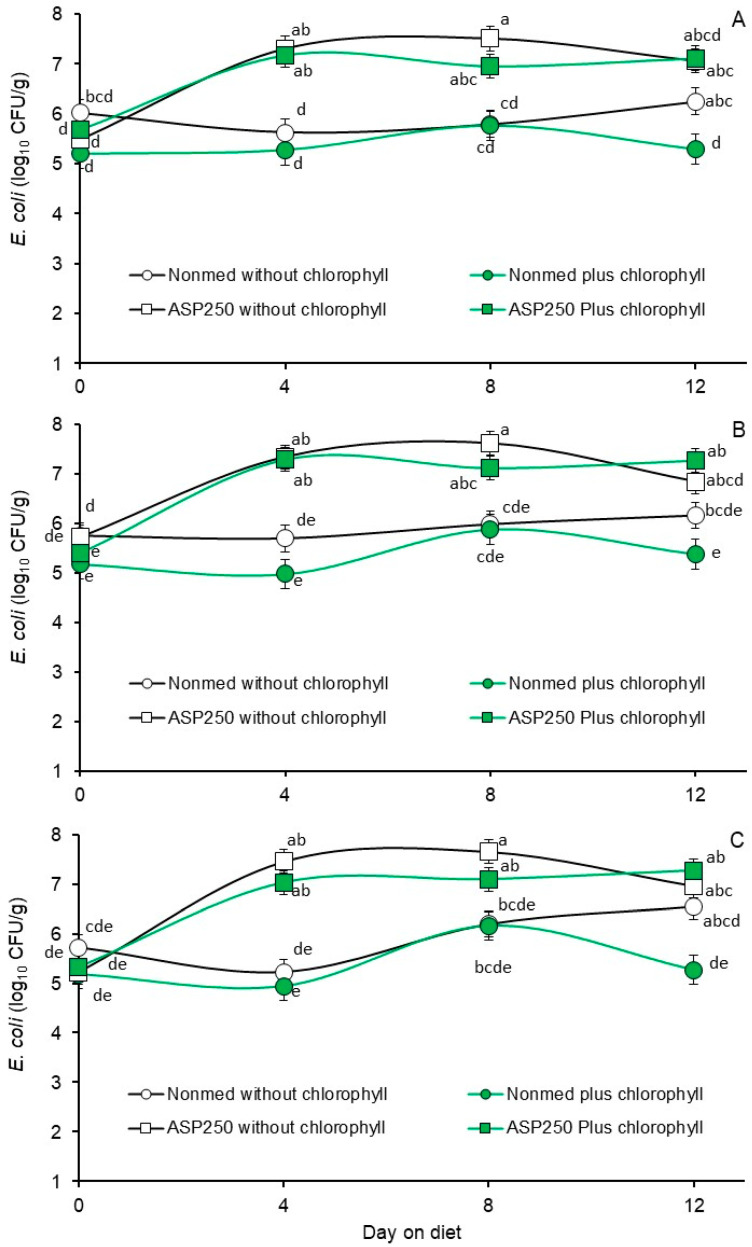
Least square means of fecal *E. coli* from the Phase 1 trial where pigs were fed non-medicated starter diets without supplemental chlorophyllin (open circles), non-medicated starter with diets supplemented with chlorophyllin (green circles), ASP 250-medicated starter diet without supplemental chlorophyllin (open squares) or ASP 250-medicated starter diet supplemented with chlorophyllin (green squares). Figure (**A**) presents *E. coli* populations enumerated on MacConkey agar containing no added antibiotics. Figures (**B**,**C**) present *E. coli* populations enumerated on MacConkey agar containing penicillin or chlortetracycline, respectively. Values are least squares means from diet by days on diet interactions based on a two-way repeated measures analysis of variance (*p* = 0.0015, 0.0009 and < 0.0001 for Figures (**A**), (**B**) and (**C**), respectively). Means within each figure associated with unlike lowercase letters differ (*p* < 0.05) as determined via the LSMEANS statement with Tukey–Kramer adjusted *p*-values and LINES option of SAS/Stat 15.1.

The main effects of diet and days on diet on fecal *E. coli* and enterococcal populations enumerated from the pigs during the Phase 1 trial are presented in Table 3 and Table 4, respectively. The main effects of diet clearly reveal that the ASP 250-medicated diet, whether without or with chlorophyllin supplementation, supported higher fecal *E. coli* numbers, and fecal *E. coli* were lowest in the non-medicated diet that had been supplemented with chlorophyllin (Table 3). The stimulatory effect of the ASP 250-medicated diets against *E. coli* is not particularly surprising as the component antibiotics, chlortetracycline and penicillin, have been extensively used in swine production. The observed interactions between diet and days on diet (Figure 1A–C, respectively) support the concept that the fecal *E. coli* may have been largely insensitive to the ASP 250 medication. For instance, the ASP 250-stimulated increase in *E. coli* populations differed little whether the *E. coli* was enumerated on non-antibiotic supplemented MacConkey agar (*p* = 0.0015) or on MacConkey agar containing 8 µg of added penicillin/mL (*p* = 0.0009) or 16 µg of added chlortetracycline/mL medium (*p* < 0.0001). A comparison of least squares means for the diet by days on diet interaction indicated that while penicillin- and chlortetracycline-insensitive *E. coli* were proportionally predominant within the pig gut upon initiation of the trial, and the *E. coli* populations were not subsequently enriched within the feces of pigs during feeding of the non-medicated diet (Figure 1). Moreover, penicillin- and chlortetracycline-insensitive *E. coli* populations were present within the gut of pigs fed the non-ASP 250-medicated diets. However, there was minimal evidence that the penicillin- and chlortetracycline-insensitive fecal *E. coli* populations recovered from the pigs fed the non-medicated diet responded differently than the *E. coli* population enumerated on non-antibiotic supplemented MacConkey agar. This suggests that the *E. coli* population enumerated on non-antibiotic supplemented MacConkey agar was predominantly composed of penicillin- and chlortetracycline-insensitive *E. coli* even before the start of the 12-day feeding trial, and these populations were unaffected by chlorophyllin supplementation. This finding is supported by high correlation coefficients (0.90 to 0.94) resulting from correlation analysis of the curves between the different recovery media. The finding that there was little effect of chlorophyllin supplementation on *E. coli* is also not unexpected, as the outer membrane of Gram-negative bacteria is considered to make them less sensitive to the effects of efflux pump inhibitors than Gram-positive bacteria [21].

**Figure 2:**
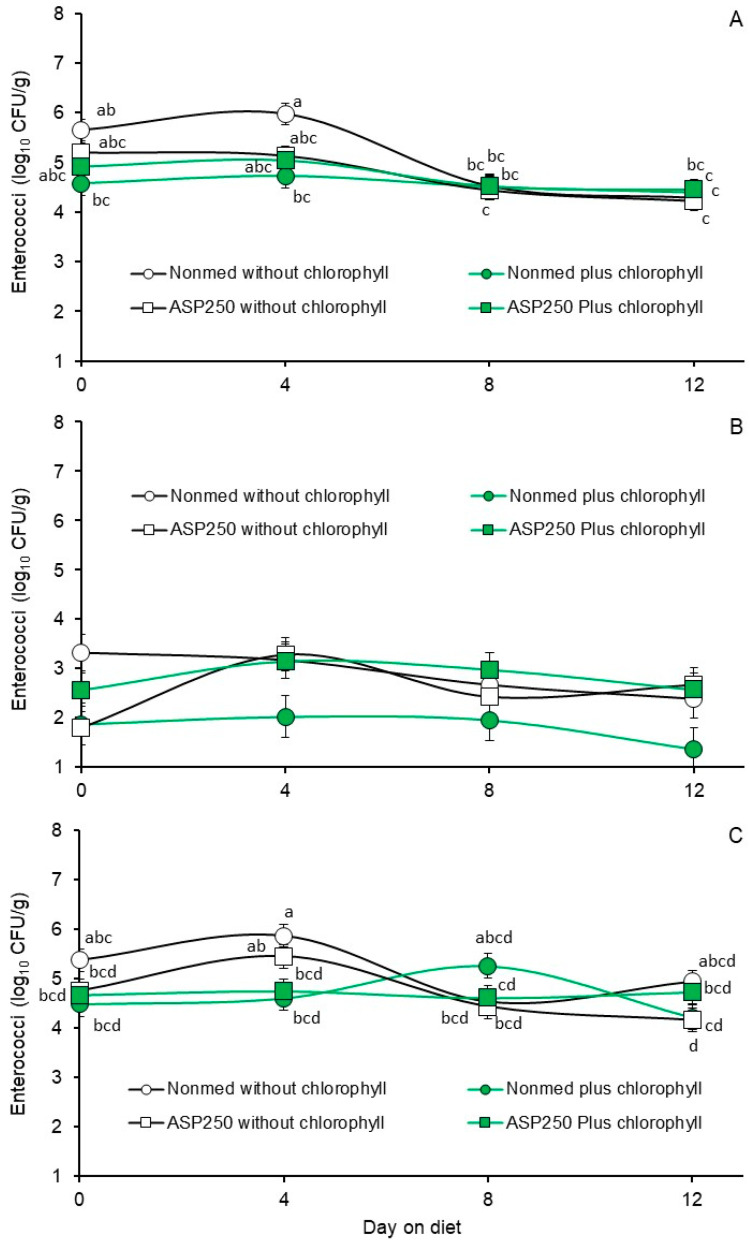
Least square means of fecal enterococci from the Phase 1 trial where pigs were fed non-medicated starter diets without supplemental chlorophyllin (open circles), non-medicated starter with diets supplemented with chlorophyllin (green circles), ASP 250-medicated starter diet without supplemental chlorophyllin (open squares) or ASP 250-medicated starter diet supplemented with chlorophyllin (green squares). Figure (**A**) presents enterococci populations enumerated on M *Enterococcus* agar containing no added antibiotics. Figures (**B**,**C**) present enterococci populations enumerated on M *Enterococcus* agar containing penicillin or chlortetracycline, respectively. Values are least squares means from diet by days on diet interactions based on a two-way repeated measures analysis of variance (*p* = 0.0511, 0.0009 and 0.1522 for Figures (**A**), (**B**) and (**C**), respectively). Means within each figure, when associated with unlike lowercase letters, differ (*p* < 0.05) or tended to differ (*p* > 0.05 < 0.10) as determined via the LSMEANS statement with Tukey–Kramer adjusted *p*-values and the LINES option of SAS/Stat 15.1.

In contrast to that observed with *E. coli*, fecal enterococci populations were generally lower in the non-medicated diet that was not supplemented with chlorophyllin than in the other diets, although the differences between treatments were not always significant (Table 3). For the fecal enterococcal populations enumerated during Phase 1, a tendency (*p* = 0.0511) for a diet by days on diet interaction was observed with populations enumerated on M *Enterococcus* agar without antibiotic selection, and a significant interaction was observed for populations enumerated on M *Enterococcus* agar containing 16 µg added/chlortetracycline/mL (*p* = 0.0009, Figure 2A–C, respectively). The findings suggest that these populations from pigs fed non-chlorophyllin-supplemented diets were transiently higher, although not statistically significant, than populations from pigs fed diets supplemented with chlorophyllin. Conversely, a significant diet and days on diet interaction was not observed for populations enumerated on M *Enterococcus* agar containing 8 µg of added penicillin/mL (*p* = 0.1522, Figure 2B). Least squares means for the main effects of diet and days on diet for the penicillin-insensitive enterococci enumerated on M *Enterococcus* agar containing 8 µg added penicillin/mL are presented in Table 3 and Table 4. The least squares means for the main effect of diet were highest for the non-medicated diet without chlorophyllin supplementation and the ASP 250-medicated diet with chlorophyllin supplementation, intermediate for the ASP 250-medicated diet without chlorophyllin supplementation highest and lowest for the non-medicated diet with chlorophyllin supplementation (Table 3). Least squares means for the main effect of time on the penicillin-insensitive enterococci during the Phase 1 trial were highest on day 4, intermediate on day 8 and lowest on days 0 and 12 (Table 4). 

Based on a comparison of least squares means for the significant or tending-significant diet by day on diet interactions (Figure 2A,C), it appears that there was little appreciable effect on the enterococcal populations regardless of whether enumerated on agar without or with added chlortetracycline. For instance, the only significant differences were the enterococcal populations recovered in feces collected from the non-medicated diets on day 4, with the populations supplemented with chlorophyllin being lower than the non-medicated populations not supplemented with chlorophyllin (Figure 2A,C). For the sake of comparison, we provide the least squares means of the nonsignificant diet by time on diet interaction for the penicillin-insensitive enterococci (Figure 2B).

### 3.3. Phase 2, Effects Non- and Tylosin-Medicated Diets without or with Chlorophyllin Supplementation on Fecal E. coli and Enterococcal Populations

**Figure 3:**
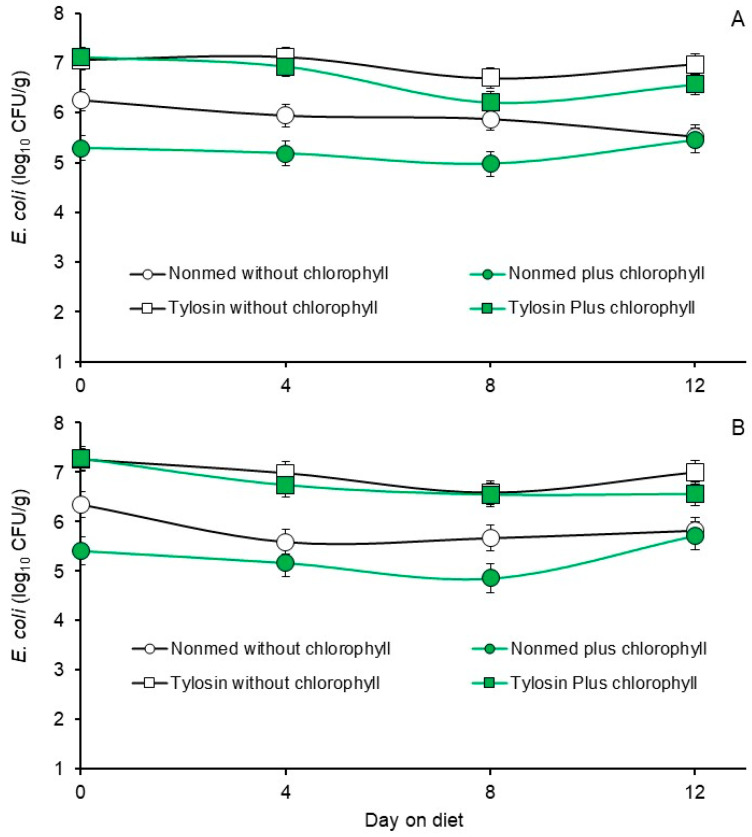
Least square means of fecal *E. coli* from the Phase 2 trial where pigs were fed non-medicated starter diets without supplemental chlorophyllin (open circles), non-medicated starter with diets supplemented with chlorophyllin (green circles), tylosin-medicated starter diet without supplemental chlorophyllin (open squares) or tylosin-medicated starter diet supplemented with chlorophyllin (green squares). Figure (**A**) presents *E. coli* populations enumerated on MacConkey agar containing no added antibiotics. Figure (**B**) presents *E. coli* populations enumerated on MacConkey agar containing tylosin. Values are least squares means from diet by days on diet interactions based on a two-way repeated measures analysis of variance (*p* = 0.2536, and 0.5530 for Figures (**A**) and (**B**), respectively). Means within each figure did not differ (*p* > 0.05) as determined via the LSMEANS statement with Tukey–Kramer adjusted *p*-values and the LINES option of SAS/Stat 15.1.

The main effects of diet and days on diet on fecal *E. coli* and enterococcal populations enumerated from the pigs during the Phase 2 trial are presented in Table 5 and Table 6, respectively. In the case of the *E. coli* populations, interactions between diet and days on diet were not observed whether the populations were enumerated on MacConkey agar containing no added antibiotic (*p* = 0.2536) or on MacConkey agar containing 100 µg added tylosin/mL (*p* = 0.5530, Figure 3). Least squares means for the main effect of diet suggest that there was an enrichment of tylosin-insensitive *E. coli* in the feces collected from pigs fed the tylosin-medicated diet as evidenced by the highest *E. coli* counts in these populations (Table 5). Populations of *E. coli* were lowest in feces from pigs fed the non-medicated diet that was supplemented with chlorophyllin and intermediate in feces from pigs fed the non-medicated diet that had not been supplemented with chlorohyllin (Table 5). This suggests that in the absence of the selective pressure of tylosin, the chlorophyllin supplementation may have exhibited a modest inhibitory effect against the *E. coli*. Least squares means for the main effect of days on diet on the all-inclusive *E. coli* were highest on days 0 and 12 and transiently lower on days 4 and 8 (Table 6). 

**Figure 4:**
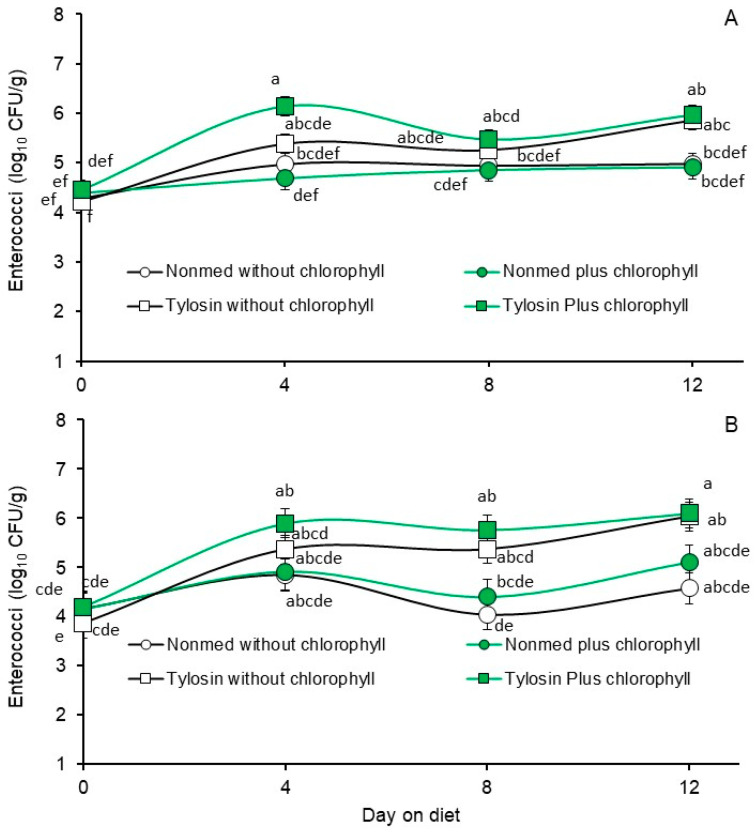
Least square means of fecal enterococci from the Phase 2 trial where pigs were fed non-medicated starter diets without supplemental chlorophyllin (open circles), non-medicated starter with diets supplemented with chlorophyllin (green circles), tylosin-medicated starter diet without supplemental chlorophyllin (open squares) or tylosin-medicated starter diet supplemented with chlorophyllin (green squares). Figure (**A**) presents enterococci populations enumerated on M *Enterococcus* agar containing no added antibiotics. Figure (**B**) presents enterococci populations enumerated on M *Enterococcus* agar containing tylosin. Values are least squares means from diet by days on diet interactions based on a two-way repeated measures analysis of variance (*p* = 0.0003 and 0.0298 for Figures (**A**) and (**B**), respectively). Means within each figure, when associated with unlike lowercase letters, differ (*p* < 0.05) as determined via the LSMEANS statement with Tukey–Kramer adjusted *p*-values and the LINES option of SAS/Stat 15.1.

In contrast with that observed with the *E. coli* populations during the Phase 2 trial, significant interactions between diet and days on diet were observed for the enterococcal populations enumerated on M *Enterococcus* agar supplemented without (*p* = 0.0003) or with 100 µg/mL added tylosin (*p* = 0.0298, Figure 4). These results suggest an enrichment, albeit modest, of tylosin-insensitive enterococci in populations recovered from feces of pigs fed the tylosin-medicated diet.

The evaluation of chlorophyll as a potential source for efflux pump inhibitors has been conducted by other groups with similar results. Specifically, the metabolically efflux pump antagonist pyropheophorbide a has yielded promising results [22]. Pyropheophorbide a also exhibited broad spectrum activity, even potentially targeting the erythromycin resistance of anaerobes enumerated via anaerobic viable cell counts [18]. Overall, chlorophyll has historically provided a number of pharmacological candidates for antibiotic development [22,23]. By potentially harnessing the microbiota to catabolize and biotransform the multiple classes of compounds found within chlorophyll pigment, an alternative to antibiotics currently supplemented in feed could potentially be developed. While the end result is the same, public appreciation for the more natural alternative will be of value to the swine industry. The use of chlorophyllin as a water-soluble chlorophyll substitute in the present study may have influenced the present results as evidenced by a lack of effect of the chlorophyllin supplementation against wild-type enterococci. Chlorophyllins may contain copper or zinc rather than magnesium in the active center, and their digestion may yield intermediate and end products structurally similar to those of natural chlorophylls with the exception that the copper or zinc cations may be retained, whereas the products of natural chlorophyll degradation no longer retain the magnesium cation [17,18]. Additionally, mechanisms of antibiotic resistance are ongoing, enabling the bacteria to overcome the antagonism to the efflux pump. Additionally, two component systems associated with efflux pumps can demonstrate an increase in antibiotic resistance depending on the antagonist potential [24]. Therefore, while an increase in resistance may seem undesirable, it could be a significant insight into the effects of the potential efflux pump antagonist as well as its use as a potential veterinary antibiotic agent. Although biological activity of chlorophyllin has been reported in several studies [14,18,25,26,27], as there is an increase in resistance in this study with some pathogens, more studies are needed. It may be important to refine and characterize the potential inhibitors from fecal extracts to evaluate the best dose regimen as well as evaluate the efflux antagonists for a potential mechanism affecting a two-component system. If the compounds are in fact potentiators, they could potentially restore susceptibility to resistant organisms [6]. All of this requires additional investigation. Furthermore, an in vivo metagenomics study may be necessary to fully understand the metagenomic impact of these compounds on the total gut microbial resistome of the pig.

## 4. Conclusions

The results from the present study indicate that feeding chlorophyll as a source or precursor of some known efflux pump inhibitors may indeed promote a decrease in antibiotic resistance, but again, the activity of these potential affecter compounds appears to be more effective against enterococci than *E. coli*. Consequently, it is unlikely at the present time that the efflux pump inhibitors tested in the present study can be developed into an inexpensive technology to preserve and enhance the efficacy of currently available antibiotics. However, further research designed to optimize chlorophyll administration could potentially lead to practical applications for the swine industry.

## Figures and Tables

**Table 1:** Diets administered to pigs during in vivo feeding studies.

	Study One (Diets) ^1^	
	Phase 1 (Days 0 to 12) 512 Power Pig Starter 30–50	Phase 2 (Days 13 to 24) 510 Power Pig Grower
Crude protein	21%	20%
Lysine	1.35%	1.26%
Crude fat	5.55%	3.80%
Crude fiber	6.00%	6.00%
Calcium	0.70 to 1.20%	0.70 to 1.20%
Phosphorus	0.65%	0.68%
Salt	0.35 to 0.85%	0.35 to 0.85%
Selenium	0.30 ppm	0.30 ppm
Zinc	250 ppm	150 ppm

^1^ Diets were purchased from Producers Cooperative Association, Bryan, TX, USA and values are guaranteed minimum concentrations unless indicated otherwise. Power Pig Starter diets medicated with ASP 250 contained 100 g chlortetracycline/ton, 50 g penicillin/ton and 100 g sulfamethazine/ton. Medicated Power Pig Grower diets contained 100 g tylosin/ton.

**Table 2:** Least squares means for performance measurements during the Phase 1 (ASP 250 medication) or Phase 2 (tylosin medication) feeding trials without or with added chlorophyllin.

	Phase 1 (ASP 250) Feeding Trial					Phase 2 (Tylosin) Feeding Trial				
Diet	Initial Body Weight (kg)	Ending Body Weight (kg) ^1^	Average Daily Feed Intake (kg/day as Fed)	Average Daily Gain (kg/day as Fed)	Feed Conversion Ratio	Initial Body Weight (kg) ^1^	Final Body Weight (kg)	Average Daily Feed Intake (kg/day as Fed)	Average Daily Gain (kg/day as Fed)	Feed Conversion Ratio
Non-medicated starter without chlorophyllin	76.7	92.9	4.25	1.35	3.32	92.9	106.3	4.36	1.12	4.04
Non-medicated starter with chlorophyllin	72.3	88.9	4.39	1.38	3.20	88.9	100.5	4.40	0.97	6.35
Medicated starter without chlorophyllin	71.0	91.4	4.15	1.71	2.47	91.4	100.7	4.28	0.98	5.86
Medicated starter with chlorophyllin	67.0	84.0	4.21	1.41	3.14	84.0	95.8	4.18	0.77	4.32
*p*-value	0.3953	0.4280	0.4981	0.1926	0.1625	0.4280	0.4281	0.0528	0.2184	0.3499
SEM	4.56	4.75	0.120	0.150	0.328	4.75	5.11	0.062	0.135	1.162

^1^ Ending body weights of the Phase 1 feeding trial are used as the initial body weights for the Phase 2 feeding trial. Values are the least squares means from the main effect of diet based on a one-way analysis of variance. Means within columns did not differ (*p* > 0.05) as determined via the LSMEANS statement with Tukey–Kramer adjusted *p*-values and the LINES option of SAS/Stat 15.1.

**Table 3:** Least squares means for the main effect of diet on fecal populations of wild-type *Escherichia coli* and enterococci recovered on MacConkey or M *Enterococcus* agars without or with additions of 8 µg penicillin/mL or 16 µg chlortetracycline/mL during the Phase 1 feeding trial.

	*Escherichia coli* (log_10_ CFU/g Feces)			Enterococci (log_10_ CFU/g Feces)		
Diet	Population Recovered without Antibiotic Selection	Population Recovered with Penicillin Selection	Population Recovered with Chlortetracycline Selection	Population Recovered without Antibiotic Selection	Population Recovered with Penicillin Selection	Population Recovered with Chlortetracycline Selection
Non-medicated starter without chlorophyllin	5.92 ^b^	5.89 ^b^	5.92 ^b^	5.22 ^a^	2.88 ^a^	5.18 ^a^
Non-medicated starter with chlorophyllin	5.38 ^c^	5.35 ^c^	5.38 ^c^	4.55 ^b^	1.80 ^b^	4.64 ^b^
ASP 250-medicated starter without chlorophyllin	6.84 ^a^	6.87 ^a^	6.82 ^a^	4.75 ^b^	2.54 ^ab^	4.70 ^b^
ASP 250-medicated starter with chlorophyllin	6.73 ^a^	6.77 ^a^	6.68 ^a^	4.73 ^b^	2.81 ^a^	4.69 ^b^
*p*-value	0.0001	0.0001	0.0001	0.0040	0.0325	0.0126
SEM	0.1264	0.1293	0.1453	0.1386	0.2903	0.1422

^abc^ Values are the least squares means from the main effect of diet based on a two-way repeated measures analysis of variance. Means within each column associated with unlike superscripts differ at *p* < 0.05 as determined via the LSMEANS statement with Tukey–Kramer adjusted *p*-values and LINES option of SAS/Stat 15.1.

**Table 4:** Least squares means for the main effect of days on diet on fecal populations of wild-type *Escherichia coli* and enterococci recovered on MacConkey or M *Enterococcus* agars without or with additions of 8 µg penicillin/mL or 16 µg chlortetracycline/mL during the Phase 1 feeding trial.

	*Escherichia coli* (log_10_ CFU/g Feces)			Enterococci (log_10_ CFU/g Feces)		
Days on Diet	Population Recovered without Antibiotic Selection	Population Recovered with Penicillin Selection	Population Recovered with Chlortetracycline Selection	Population Recovered without Antibiotic Selection	Population Recovered with Penicillin Selection	Population Recovered with Chlortetracycline Selection
0	5.60 ^b^	5.51 ^b^	5.36 ^c^	5.08 ^a^	2.39 ^b^	4.82 ^b^
4	6.35 ^a^	6.32 ^a^	6.16 ^b^	5.21 ^a^	2.90 ^a^	5.17 ^a^
8	6.50 ^a^	6.64 ^a^	6.77 ^a^	4.50 ^b^	2.50 ^ab^	4.71 ^bc^
12	6.43 ^a^	6.41 ^a^	6.51 ^a^	4.45 ^b^	2.25 ^b^	4.51 ^c^
*p*-value	0.0001	0.0001	0.0001	0.0001	0.0227	0.0007
SEM	0.1326	0.1273	0.1294	0.1107	0.1851	0.1108

^abc^ Values are the least squares means from the main effect of days on diet based on a two-way repeated measures analysis of variance. Means within columns associated with unlike superscripts differ at *p* < 0.05 as determined via the LSMEANS statement with Tukey–Kramer adjusted *p*-values and LINES option of SAS/Stat 15.1.

**Table 5:** Least squares means for the main effect of diet on fecal populations of wild-type *Escherichia coli* and enterococci recovered on MacConkey or M *Enterococcus* agars without or with the addition of 100 µg tylosin/mL during the Phase 2 feeding trial.

	*Escherichia coli* (log_10_ CFU/g Feces)		Enterococci (log_10_ CFU/g Feces)	
Diet	Population Recovered without Antibiotic Selection	Population Recovered with Tylosin Selection	Population Recovered without Antibiotic Selection	Population Recovered with Tylosin Selection
Non-medicated starter without chlorophyllin	5.90 ^b^	5.86 ^b^	4.91 ^bc^	4.40 ^c^
Non-medicated starter with chlorophyllin	5.22 ^c^	5.28 ^c^	4.71 ^c^	4.64 ^bc^
Tylosin-medicated starter without chlorophyllin	6.96 ^a^	6.95 ^a^	5.19 ^ab^	5.15 ^ab^
Tylosin-medicated starter with chlorophyllin	6.70 ^a^	6.78 ^a^	5.51 ^a^	5.49 ^a^
*p*-value	0.0001	0.0001	0.0011	0.0021
SEM	0.1633	0.1848	0.1572	0.2399

^abc^ Values are the least squares means from the main effect of diet based on a two-way repeated measures analysis of variance. Means within columns associated with unlike lowercase letters differ (*p* < 0.05) as determined via the LSMEANS statement with Tukey–Kramer adjusted *p*-values and LINES option of SAS/Stat 15.1.

**Table 6:** Least squares means for the main effect of days on diet on fecal populations of wild-type *Escherichia coli* and enterococci recovered on MacConkey or M *Enterococcus* agars without or with the addition of 100 µg tylosin/mL during the Phase 2 feeding trial.

	*Escherichia coli* (log_10_ CFU/g Feces)		Enterococci (log_10_ CFU/g Feces)	
Days on Diet	Population Recovered without Antibiotic Selection	Population Recovered with Tylosin Selection	Population Recovered without Antibiotic Selection	Population Recovered with Tylosin Selection
0	6.43 ^a^	6.57 ^a^	4.45 ^c^	4.09 ^c^
4	6.29 ^a^	6.12 ^bc^	5.30 ^ab^	5.25 ^ab^
8	5.94 ^b^	5.91 ^c^	5.13 ^b^	4.89 ^b^
12	6.13 ^ab^	6.27 ^ab^	5.43 ^a^	5.45 ^a^
*p*-value	0.0088	0.0014	0.0001	0.0001
SEM	0.1097	0.1268	0.1021	0.1592

^abc^ Values are the least squares means from the main effect of days on diet based on a two-way repeated measures analysis of variance. Means within columns associated with unlike lowercase letters differ (*p* < 0.05) as determined via the LSMEANS statement with Tukey–Kramer adjusted *p*-values and the LINES option of SAS/Stat 15.1. Means with unlike superscripts differ at *p* < 0.05.

## Data Availability

Data generated or analyzed during this study are available from the corresponding author upon reasonable request.

## References

[1] Angulo F.J., Baker N.L., Olsen S.J., Anderson A., Barrett T.J. (2004). Antimicrobial use in agriculture: Controlling the transfer of antimicrobial resistance to humans. Sem. Pediatric Infect. Dis..

[2] Gorbach S.L. (2001). Antimicrobial use in animal feed—Time to stop. N. Engl. J. Med..

[3] McEwen S.A., Fedorka-Cray P.J. (2002). Antimicrobial use and resistance in animals. Clin. Infect. Dis..

[4] Looft T., Johnson T.A., Allen H.K., Bayles D.O., Alt D.P., Stedtfeld R.D., Sul W.J., Stedtfeld T.M., Chai B., Cole J.R. (2012). In-feed antibiotic effects on the swine intestinal microbiome. Proc. Nat. Acad. Sci. USA.

[5] Wu F., Tokach M.D., DeRouchey J.M., Dritz S.S., Woodworth J.C., Goodband R.D., Chitakasempornkul K., Bello N.M., Capps K., Remfry S. (2019). Effects of tylosin administration routes on the prevalence of antimicrobial resistance among fecal enterococci of finishing swine. Foodborne Pathog. Dis..

[6] Pagès J., Masi M., Barbe J. (2005). Inhibitors of efflux pumps in Gram-negative bacteria. Trends Mol. Med..

[7] Starvi M., Piddock L.J.V., Gibbons S. (2007). Bacterial efflux pump inhibitors from natural sources. J. Antimicrob. Chemother..

[8] Nikaido H. (1998). The role of outer membrane and efflux pumps in the resistance of gram-negative bacteria. Can we improve drug access?. Drug Resist. Updat..

[9] Poole K. (2007). Efflux pumps as antimicrobial resistance mechanisms. Ann. Med..

[10] Sáenz Y., Ruiz J., Zarazaga M., Teixido M., Torres C., Vila J. (2004). Effect of the efflux pump inhibitor phe-arg-β-napththylamide on the MIC values of the quinolones, tetracyclines and chloramphenicol, in *Escherichia coli* isolates of different origin. J. Antimicrob. Chemother..

[11] Stermitz F.R., Tawara-Matsuda J., Lorenz P., Mueller P., Zenewicz L., Lewis K. (2005). 5′-Methoxyhydnocarpin-D and pheophorbide A: *Berberis* species components that potentiate berberine growth inhibition of resistant *Staphylococcus aureus*. J. Nat Prod..

[12] Tegos G., Stermitz F.R., Lomovskaya O., Lewis K. (2002). Multidrug pump inhibitors uncover remarkable activity of plant antimicrobials. Antimicrob. Agents Chemother..

[13] Barnes C.B., Rasmussen S.L., Petrich J.W., Rasmussen M.A. (2012). Determination of the concentration of potential efflux pump inhibitors, pheophorbide a and pyropheophorbide a, in the feces of animals by fluorescence spectroscopy. J. Agric. Food Chem..

[14] Ferruzzi M.G., Blakeslee J. (2007). Digestion, absorption, and cancer preventative activity of dietary chlorophyll derivatives. Nutr. Res..

[15] Ferruzzi M.G., Failla M.L., Schwartz S.J. (2002). Sodium copper chlorophyllin: In vitro digestive stability and accumulation by Caco-2 Human intestinal cells. J. Agric. Food Chem..

[16] Zhong S., Bird A., Kopec R.E. (2021). The metabolism and potential bioactivity of chlorophyll and metallo-chlorophyll derivatives in the gastrointestinal tract. Mol. Nutr. Food Res..

[17] Pérez-Gálvez A., Viera I., Benito I., Roca M. (2020). HPLC-hrTOF-MS study of copper chlorophylls: Composition of food colorants and biochemistry after ingestion. Food Chem..

[18] Tumulo T., Lanfer-Marquez U.M. (2012). Copper chlorophyllin: A food colorant with bioactive properties?. Food Res. Int..

[19] Martins C.F., Lopes P.A., Palma M., Pinto R.M.A., Costa M., Alfaia C.M., Pestana J.M., Coelho D., Ribeiro D.M., Viegas I. (2022). Impact of dietary *Chlorella vulgaris* and feed enzymes on health status, immune response and liver metabolites in weaned piglets. Sci. Rep..

[20] Martins C.F., Pestana Assunção J., Ribeiro Santos D.M., Madeira M.S.M.D.S., Alfaia C.M.R.P.M., Lopes P.A.A.B., Coelho D.F.M., Lemos J.P.C., de Almeida A.M., Prates J.A.M. (2021). Effect of dietary inclusion of Spirulina on production performance, nutrient digestibility and meat quality traits in post-weaning piglets. J. Anim. Physiol. Anim. Nutr..

[21] Krüger M., Richter P., Strauch S.M., Nasir A., Burkovski A., Antunes C.A., Meißgeier T., Schlücker E., Schwab S., Lebert M. (2019). What an *Escherichia coli* mutant can teach us about the antibacterial effect of chlorophyllin. Microorganisms.

[22] Kraatz M., Whitehead T.R., Cotta M.A., Berhow M.A., Rasmussen M.A. (2014). Effect of chlorophyll-derived efflux pump inhibitor pheophorbide a and pyropheophorbide a on growth and macrolide antibiotic resistance of indicator and anaerobic swine manure bacteria. Int. J. Antibiotics..

[23] Jørgensen E.G. (1962). Antibiotic substances from cells and culture solutions of unicellular algae with special reference to some chlorophyll derivatives. Physiol. Plant..

[24] Lee A., Mao W., Warren M., Mistry A., Hoshino K., Okumura R., Ishida H., Lomovskaya O. (2000). Interplay between efflux pumps may provide either additive or multiplicative effects on drug resistance. J. Bact..

[25] de Vogel J., Jonker-Termont D.S., Katan M.B., van der Meer R. (2005). Natural chlorophyll but not chlorophyllin prevents heme-induced cytotoxic and hyperproliferative effects in rat colon. J. Nutr..

[26] Zepka L.Q., Jacob-Lopes E., Roca M. (2019). Catabolism and bioactive properties of chlorophylls. Curr. Opin. Food Sci..

[27] Zheng H., You Y., Hua M., Liu Y., Chen Z., Zhang L., Wei H., Li Y., Luo M., Zeng Y. (2018). Chlorophyllin modulates gut microbiota and inhibits intestinal inflammation to ameliorate hepatic fibrosis in mice. Front. Physiol..

